# The Effect of Ultraviolet Light Irradiation on Pigment Performance in Microwave-Assisted Extraction of *Arthrospira platensis*

**DOI:** 10.3390/md23100391

**Published:** 2025-09-30

**Authors:** Anna Trubetskaya, Roland Haseneder, Maximilian Lippold, Rob J. F. van Haren, Volker Herdegen, Lisa Ditscherlein, James J. Leahy, Italo Pisano, Yvonne Joseph, Carla Vogt, Jan Zuber

**Affiliations:** 1 Department of Biosciences, Nord University, 07713 Steinkjer, Norway; 2 Department of Chemical Sciences, University of Limerick, V94 T9PX Castletroy, Ireland; j.j.leahy@ul.ie (J.J.L.);; 3 Institute of Thermal-, Environmental- and Resources’ Process Engineering (ITUN), TU Bergakademie Freiberg, 09599 Freiberg, Germany; 4 Institute of Nanoscale and Biobased Materials, TU Bergakademie Freiberg, 09599 Freiberg, Germany; 5 Centre of Applied Research Biobased Economy, Hanze University of Applied Sciences, 9747 AS Groningen, The Netherlands; 6 Institute of Mechanical Process Engineering and Mineral Processing, TU Bergakademie Freiberg, 09599 Freiberg, Germany; 7 Institute of Analytical Chemistry, TU Bergakademie Freiberg, 09599 Freiberg, Germanyjan.zuber@chemie.tu-freiberg.de (J.Z.)

**Keywords:** biopterin–pentoside complexes, *Arthrospira platensis*, stability, UV light degradation, temperature, phycocyanin, protein interactions, FT-ICR-MS

## Abstract

Phycocyanin, a blue pigment from *Arthrospira platensis*, is widely used as a natural colorant in food products, but its application is limited by its sensitivity to light and temperature during extraction and storage. This study explored the impact of UV light on phycocyanin extracted from *A. platensis* using a microwave-assisted method. Water proved to be the most effective solvent, yielding the highest phycocyanin concentration and stability. The optimal extraction conditions to avoid phycocyanin degradation were identified as 45 °C and 100 W of microwave power. Fourier transform ion cyclotron resonance mass spectrometry (FT-ICR-MS) analysis revealed increased chemical complexity at higher temperatures and identified biopterin–pentoside complexes, which enhanced phycocyanin stability during UV degradation. These findings provide new insights into the molecular mechanisms of interactions between phycocyanin and proteins, enhancing phycocyanin stability and functionality and thus providing food products with longer shelf lives by maintaining their nutritional and aesthetic qualities.

## 1. Introduction

Phycobiliproteins are the main photosynthetic accessory pigments in *A. platensis (Gomont)*, a non-toxic cyanobacterium. *A. platensis* cells have shown resilience when exposed to harsh pH conditions (>10); UV radiation (>0.6 mW m^−2^); and high concentrations of bicarbonate (HCO_3_^−^), a key inorganic carbon source that supports their photosynthetic activity and adaptation to alkaline environments [1,2,3]. Despite phycobiliproteins being linked to the outer surfaces of photosynthetic cell lamellae, the chromophore in the blue-colored phycocyanin, which comprises the α-subunit (20.5 kDa) and β-subunit (23.5 kDa), is sensitive to UV light [4]. Changes in chromophore sensitivity were previously related to protein conformation in *A. platensis*, leading to a lack of light stability and high sensitivity to heat at temperatures above 45 °C and under highly acidic conditions [5]. This could cause limitations in the application of phycobiliproteins in the food sector, due to fast discoloration of food products and degradation of their nutritional properties. In addition, the instability of phycobiliproteins under adverse conditions can shorten the shelf life of food products containing these pigments, leading to unnecessary food waste. Lastly, their sensitivity to environmental conditions can limit the use of phycobiliproteins in various food processing systems that involve heat or acidic environments.

Phycobiliproteins are water-soluble proteins bearing covalently attached open-chain tetrapyrroles and, therefore, they can be easily isolated as protein pigment complexes using homogenization, sonication, microwave treatments, supercritical fluid extraction, and lysozyme disintegration [6,7,8]. Phycocyanin, for example, has been extracted from cyanobacteria by different methods requiring continuous control of the pH and ionic strength to maintain complex stability [9]. New regulations and policies require the extraction process to be effective in terms of a high extraction yield and environmental sustainability [10]. Single-stage, low-energy microwave extraction of algae showed key advantages compared to conventional extraction processes in its short conversion time, product uniformity, and selective heating over the entire material within broad temperature and pressure ranges [11]. Microwave-assisted extraction utilizes microwaves (100 MHz–300 GHz) to heat an extraction medium containing solvents to isolate most of the useful chemical contents in a biological material [12]. Microwave treatments have mostly been applied to marine microalgae to extract lipids and oil [13,14,15]. However, the different studies mentioned above have suggested that microwaves could be an option for extracting phycobiliproteins from microalgae. Temperatures between 30 and 60 °C, under atmospheric pressure and vacuum microwave conditions and acetone as the extraction medium were selected for the extraction of pigments (chlorophyll *a* and fucoxanthin) from *Cylindrotheca Closterium* and a broader range of pigments (chlorophyll *a*, chlorophyll *b*, and β,β-carotene) from *Dunaliella tertiolecta* microalgae [16]. Microwave-assisted (MAE) and ultrasound-assisted (UAE) extraction methods were compared in terms of the purity and yields of C-phycocyanin from *A. platensis* [17]. The combination of various buffers (acetate at pH = 5; phosphate at pH = 6–7; and tris-Cl at pH = 8) at a frequency of 20 kHz and power of 100 W resulted in the highest concentration and purity of C-phycocyanin. Another study showed that the use of 1.5% CaCl_2_ (w/v) as the extraction medium at 40 °C delivered a higher total phycocyanin content (55 mg g^−1^) from frozen *A. platensis* cells compared to distilled water and Na-Phosphate (pH = 7.4) from dried and fresh *A. platensis* matrices [18]. In subsequent work, microwave extraction of the marine diatom *Phaeodactylum tricornutum* resulted in higher yields of fucoxanthin pigment (approximately 6 mg g^-1^), with higher antioxidant activity (EC_50_: 760 μg mL^−1^ of extract) than ultrasound extraction [19]. The pre-treatment of *A. platensis* cells using various disruption methods, e.g., ball milling, autoclaving, drying, etc. had a large impact on the yields of phycocyanin after microwave extraction at power levels up to 1400 W and frequencies from 100 to 2.450 MHz [20]. A recent review paper recommended using moderate temperatures (up to 50 °C), neutral pH varying from 6 to 8, and reduced contact with light sources for phycocyanin extraction from *A. platensis* [9]. The drawback of the microwave extraction of microalgae is the use of relatively high temperatures (>45 °C), which can result in low yields and discoloration of phycocyanin [21]. In general, microwave-assisted treatment is used for the isolation of value-added compounds at temperatures above 35 °C due to the instability of microwave power control at lower temperatures [22]. More investigations are required to understand the structural relationship between phycocyanin properties and the operating conditions of microwave extraction, as phycocyanin in *A. platensis* remains sensitive to light, selected solvents, microwave power, and temperature [23]. In recent years, scientists, policy makers, industries, and active citizens have focused on natural resources that can be an alternative to synthetic colorants for sustainable and circular use in the food sector. Phycocyanin extracted from *A. platensis* is widely recognized as safe and non-toxic for use in food products, underpinning its suitability as a natural colorant [24,25]. However, the use of phycocyanin as an organic colorant can readily lead to changes in its stability and strength in various food products, e.g., smoothies, beverages, ice cream, and dairy products, when the operating conditions of the microwave-assisted extraction are not optimized [26]. 

It is possible to prevent the discoloration of phycocyanin with the addition of biopterin-α-glucoside, emphasizing the importance of this fluorescent substance in stabilization against UV light when the extraction temperature does not exceed 80 °C [27,28]. Various solvents can be used in the microwave extraction of phycocyanin to control the strength of the pigment’s color. 

The objective of this study is to optimize extraction conditions in terms of yield and stability of *A. platensis*. Another objective is to understand the discoloration of phycocyanin under UV irradiation following microwave treatment by identifying the chemical composition using Fourier transform–ion cyclotron resonance–mass spectrometry (FT-ICR-MS). This study is the first to investigate the combined effect of UV irradiation and microwave-assisted extraction on the stability and chemical complexity of phycocyanin from *A. platensis*. By identifying biopterin–pentoside complexes through FT-ICR-MS, it introduces a novel molecular mechanism for enhancing pigment stability under UV exposure.

## 2. Results

### 2.1. Characterization of Arthrospira platensis

The ash, water, protein, and carbohydrate, and compositional analyses of the dried *A. platensis* sample are shown in Table 1.

Microalgae are known for their high ash content. In this study, the macro minerals (K, Na, Ca, Mg, P, S, Cl) were found to be high, totaling 87,400 mg kg^−1^, primarily due to the high sulfur content (27,200 mg kg^−1^), chlorine (17,200 mg kg^−1^), and phosphorus (14,000 mg kg^−1^). The high content of Ca (11,600 mg kg^−1^) in *A. platensis* harvested in Schleswig-Holstein is attributed to its bioaccumulation capabilities. The Baltic Sea basin is rich in calcium primarily due to the erosion of carbonate-bearing rocks and the influx of riverine inputs (CaCO_3_) into the water and coastal soil [29]. The natural environment of the Baltic Sea contains more Ca than Al, which is present as aluminosilicate in water and soil. In this study, the Al content (20,600 mg kg^−1^) was higher than expected due to the cultivation environment rather than the natural water composition of the region. The nitrogen content determination supported the calculation of the total protein concentration in *A. platensis*, which was 64.7 g/100 g. The carbohydrate content was determined and recalculated by AGROLAB to show the high content of proteins in *A. platensis* compared to other lignocellulosic materials. 

Microalgae naturally accumulate both minerals and heavy metals from their aqueous environment. *A. platensis* showed a low total content of heavy metals (As, Cd, Hg, Pb), < 10 mg kg^−1^, which is below EU standards, making *A. platensis* a promising candidate for use in the food sector. Additionally, the low iodine content (900 mg kg^−1^) gives *A. platensis* an advantage over seaweed in food manufacturing. The high phosphorus (14,000 mg kg^−1^) and iron (10,000 mg kg^−1^) concentrations provide *A. platensis* with properties, which are beneficial for circular and sustainable biorefinery processes, as use of high phosphorus renewable feedstocks followed by the efficient recovery of phosphorus and iron could benefit agriculture and the overall green-blue economy in Europe. Thus, pigment extraction from *A. platensis* can support the customization of microalgae for use in food, agriculture, medicine, and biomedicine, enhancing the overall value of the extracted pigment within the circular bioeconomy.

### 2.2. pH and Conductivity

**Figure 1:**
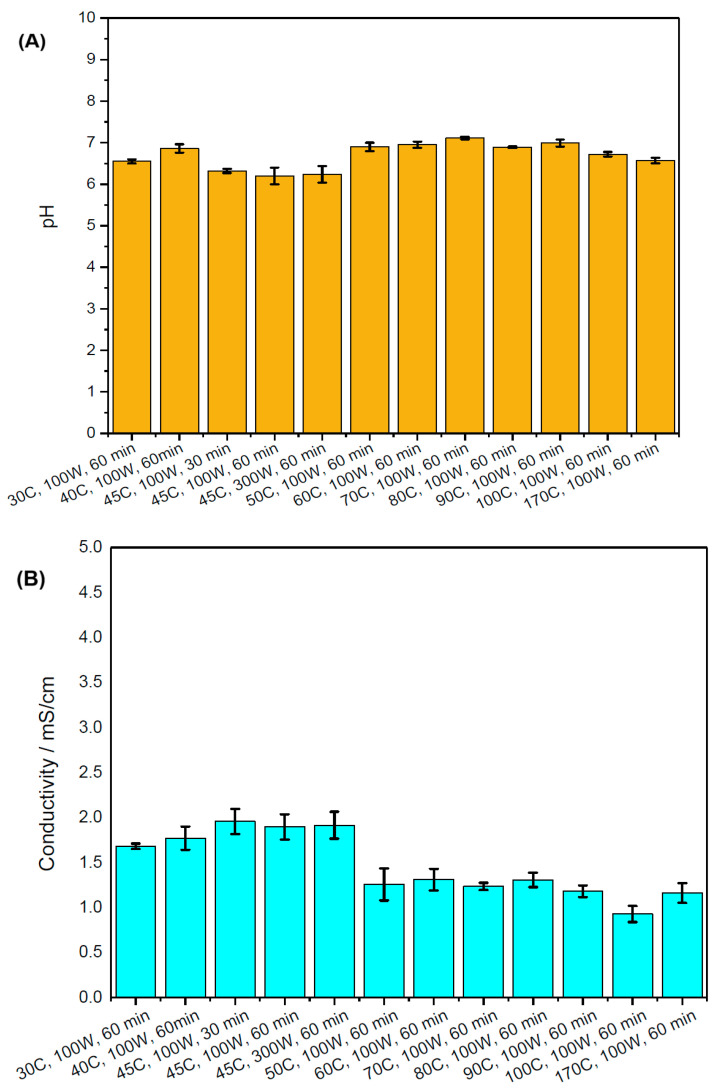
pH (**A**) and conductivity (**B**) of microwave-treated *A. platensis* at different temperatures, power, and residence time in aqueous solution.

**Figure 2:**
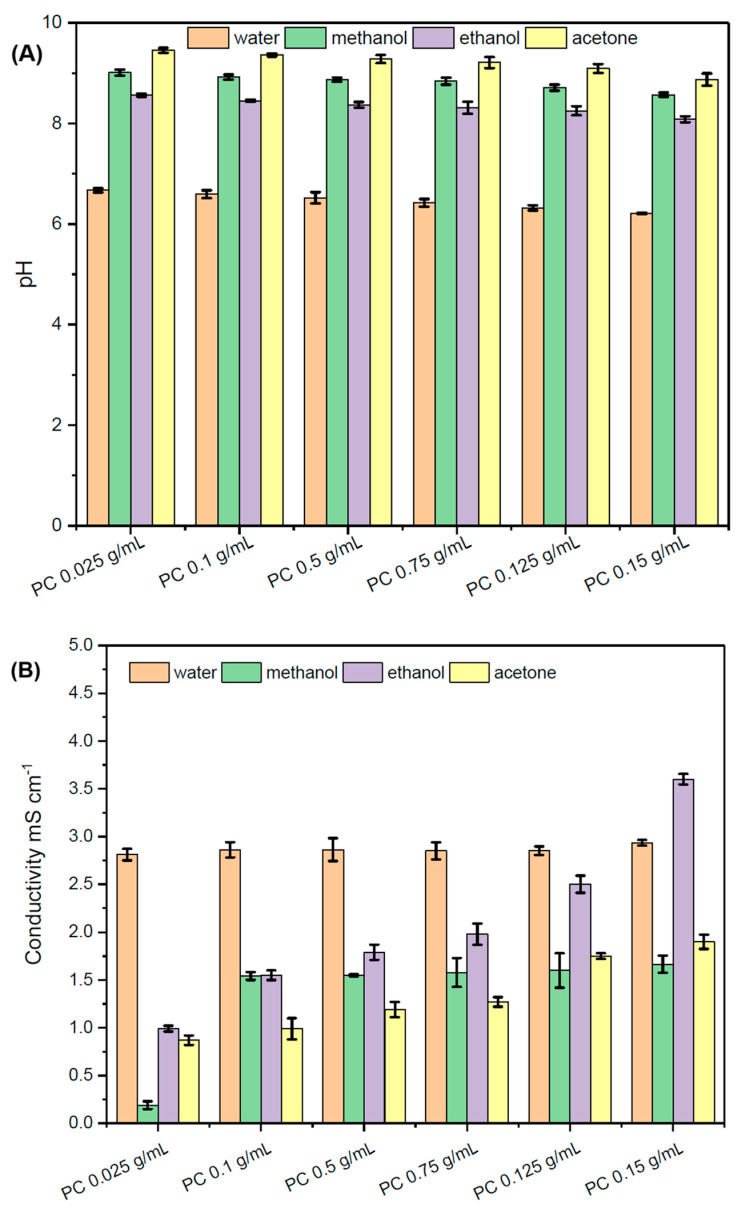
pH (**A**) and conductivity (**B**) of phycocyanin in different solvents.

Figure 1 shows the differences in pH and conductivity between aqueous extracts from microwave treatment of *A. platensis*. The main changes were observed for the microwave treatment of microalgae above 50 °C. They were more related to the conductivity than to the pH values, which remained between 6 and 8 for the entire temperature range of the microwave treatment (see Figure 1A). Despite intracellular pH being an important parameter influencing the proteins and pigment’s structure and their function, in this study, the fluorescence intensity of the aqueous phycocyanin solution had significantly less impact on the color changes, confirming previous results [30]. Figure 2 shows the impact of solvents on the pH of the phycocyanin-containing solutions, which were primarily used in the calibration of a UV-Vis instrument (see Section 4.4). The pH of phycocyanin samples in ethanol, methanol, and acetone varied between 8 and 9.5, corresponding to the maximum production of phycobiliprotein of *A. platensis* in aquatic and terrestrial plants [31]. 

This is due to the increase in hexamers at pH > 8, which increases the energy required for photosynthesis [32]. A significant increase in the percentage of α-helices and β-turns and a decrease in the percentage of single-chain protein segments can be assumed for phycocyanin solutions in ethanol, acetone, and methanol. Although the variations in pH were relatively minor, the pigment solution in acetone consistently exhibited the highest pH levels across different concentrations of phycocyanin. 

Figure 1B shows that below 45 °C, the electrical conductivity of liquid *A. platensis* extract after aqueous microwave treatment varied between 1.6 and 2 mS cm^−1^, corresponding to previous results for *A. platensis* extraction with pulsed electric treatment [33]. The differences in residence time and microwave power did not have a strong impact on the electrical conductivity of the *A. platensis* liquid extract. Microwave treatment at temperatures above 50 °C resulted in a decrease in the electric conductivity from 1.9 to 0.8 mS cm^−1^. 

Figure 1 shows that the electrical conductivity decrease can potentially be linked to the slightly increased pH values in aqueous solution. There is no direct relationship between the pH and conductivity. However, the presence of hydronium and hydroxyl ions in the extract solution is known to have an impact on its conductivity [34]. Figure 2A shows that the decrease in electrical conductivity slightly reduces the ionic strength of *A. platensis* extracts, but it does not lead to the strong discoloration of aqueous solutions. Figure 2B shows that the electrical conductivity of phycocyanin standards of different concentrations in ethanol, methanol, and acetone solvents dropped from approximately 2 mS cm^−1^ to 0.1 mS cm^−1^. 

The aqueous environment ionizes and generates free ions around phycocyanin pigments, which enhance electric conductivity, while acetone, ethanol, and methanol belong to non-electrolyte solutions, making the *A. platensis* extracts less conductive. The differences in electrical conductivity between the solvents were negligible; however, they were significant relative to the aqueous phycocyanin standards. Figure 2 specifically shows that the electrical conductivity of phycocyanin standards in methanol dropped to approximately 0.1 mS cm^−1^, comparable to the values observed in ethanol and acetone. In this study, the electrical conductivity of Millipore water was measured as 0.058 μS cm^−1^. The electrical conductivity of an aqueous pigment environment might be higher due to the presence of bioactive by-products from the extraction, as suggested previously [35].

### 2.3. Solvent and Temperature Impact on Pigments

**Figure 3:**
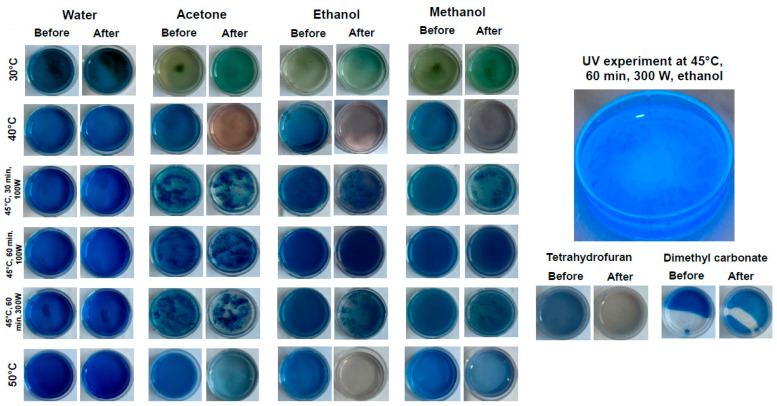
Photos of extracts from microwave treatment in a range of 30–100 °C before and after UV light exposure in water, ethanol, acetone, methanol, and microwave treatment at 50 °C in tetrahydrofuran and dimethyl carbonate.

Figure 3 illustrates the UV treatment of pigment containing extracts after microwave treatment in water, acetone, ethanol, and methanol. 

The color of the extracts only slightly changed for the samples from the microwave treatment in water, whereas acetone showed separation in two phases. The solid phase of the acetone-containing extract could include proteins that were not removed during the membrane separation and rotary evaporation. The formation of monomeric structures from protein trimers and phycocyanin conformations, caused by β-subunits unfolding or dissociation, resulted in the weaking of hydrogel bonds, hydrophobic forces, and van der Waals forces during exposure to UV light [36]. This could lead to protein aggregation and discoloration of the liquid extract phase [37,38]. 

The extracts, which were prepared in ethanol and methanol using the microwave treatment at 45 °C and power of 100 W, changed their color only slightly, emphasizing the importance of the microwave power tuning on the phycocyanin’s quality. The lower microwave power does not cause significant changes in the interactions between protein and phycocyanin, whereas the increase in power from 100 to 300 W using ethanol and methanol as solvents seems to impact the color stability due to the protein unfolding. A shift from royal dark blue to a blueish green color has been related to the further dissociation of pigments into monomers and potentially a predominance of allophycocyanin over C-phycocyanin (CPC) with increasing temperature [39]. The microwave treatment at temperatures >50 °C led to phycocyanin discoloration without forming a solid protein phase, as it was observed at lower temperatures. Compared to all other extracts, the sample in ethanol showed the complete loss of blue color without the presence of aggregates or other types of visible solid particles. The microwave treatment in tetrahydrofuran led to a similar outcome as that observed for the ethanol-containing extract. The microwave experiment with DMC as a solvent led to only a slight color change with the separation of solvent and extract phases due to its non-polarity. Figure 3 demonstrates that only the extracts from aqueous microwave treatment caused minimal changes in the blue pigment color over a broad temperature range.

### 2.4. Fluorescence Microscopy

Images of extracts in acetone, ethanol, and methanol were recorded using a fluorescence microscope. The structures with a bright green color (blue excitation: 420–500 nm) in extracts from microwave treatment at 30 °C in ethanol and methanol, and later in all three solvents at 60 °C, were related to the presence of lipids [40,41]. The fluorescence measurements are shown for the extracts from microwave treatment at temperatures below 60 °C because the extracts from microwave extraction at higher temperatures did not show any differences over the entire range of wavelengths. This corroborates previous results on extraction from *A. platensis*, underlying the preferences for low microwave treatment temperatures to minimize changes to the structure and properties of phycocyanin [42].

**Figure 4:**
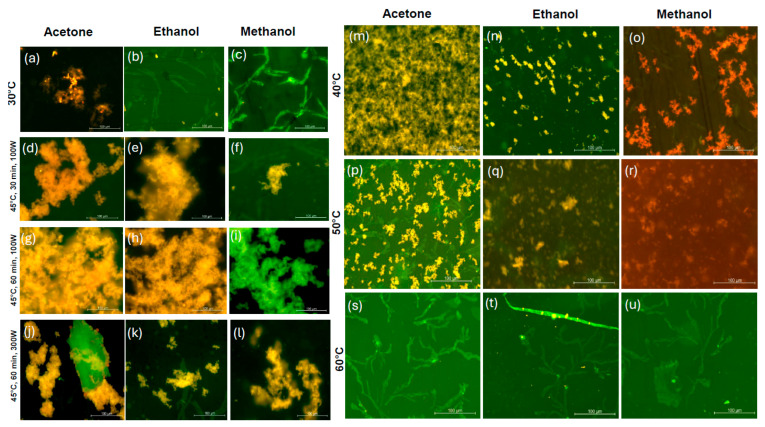
Fluorescence microscopy of extracts from microwave treatment at 30–60 °C in acetone, ethanol, and methanol (**a**–**u**).

Yellow-orange fluorescence, as shown in Figure 4d–h,j–n,p,q, indicates the presence of lipids or C-/R-phycoerythrin (green excitation: 510–560 nm, 570–575 nm) [43]. Figure 4o,r display weak red fluorescence, which is hypothesized to be from allophycocyanin, which typically emits at longer wavelengths (650 nm) than C-phycocyanin (617 nm) [44]. Both C-phycocyanin (630–650 nm) and R-phycocyanin (635–638 nm) pigments are expected to emit a strong red fluorescence [45]. In this work, it is possible that different types of phycocyanin and phycoerythrin emitted a mixture of yellow, orange, and red fluorescence in methanol. Compared to the yellow fluorescence of extracts from microwave treatment at 40 and 50 °C in acetone and ethanol in Figure 4m,n,p,q, this highlights the importance of the solvent selection on the properties and composition of *A. platensis* pigments. In addition, a mixture of green and yellow fluorescence is shown in Figure 4i,j. 

Despite the preparation of all images using the same measurement bar in fluorescence microscopy, the microwave treatment at 50 °C caused the fluorescence-emitting pigments to form smaller sized aggregates with other protein extracts with different solvents. 

### 2.5. UV-Vis Spectroscopy

The results from fluorescence microscopy were complemented with the UV-Vis characterization, followed by the calculation of the phycocyanin’s concentrations in all solvents, as shown in Table 2. The extract from microwave treatment in water showed the highest yield of phycocyanin of 3.1 mg mL^−1^. The concentration of phycocyanin decreased during microwave treatment at temperatures ranging from 30 to 70 °C. A significant decrease in phycocyanin yield of more than seven times occurred at 50 °C. The differences in phycocyanin yield among extracts in water, acetone, methanol, and ethanol were negligible at microwave temperatures above 30 °C.

The UV-Vis measurements of the microwave extracts in water and ethanol show maxima at 340 nm and 414 nm, which belong to chlorophyll and carotenoids, while the maximum at 665 nm is characteristic of allophycocyanin [45,46]. These maxima were unnoticeable in methanol and acetone. This can be attributed to the solvent polarity and its impact on pigment solubility and chromophore integrity. Methanol and acetone, being less favorable for stabilizing phycocyanin’s native conformation, is likely to promote pigment dissociation or degradation under UV exposure. Additionally, the lack of visible maxima suggests that these solvents may interfere with the electronic transitions of chlorophyll and carotenoids, reducing their detectability in the UV-Vis spectrum. This highlights the importance of solvent selection not only for extraction efficiency but also for preserving spectral characteristics essential for pigment identification and quantification.

### 2.6. Chemical Composition of Aqueous Pigments Using FT-ICR-MS Analyses

The GALDI-FT-ICR-MS analyses of the *A. platensis* samples, which were prepared at 40 °C and 170 °C for 60 min using 100 W of power, showed that more compounds could be ionized by applying the negative ion mode (GALDI(−)) in comparison to the positive ion mode (GALDI(+)). This is especially illustrated by the peak numbers, which were 2926 for the 40 °C liquid extract and 2,946 for the 170 °C sample using GALDI(−)-MS and 1769 (40 °C)/2350 (170 °C) for GALDI(+)-MS. Hence, regardless of the mode of ionization, more compounds could be ionized in the 170 °C sample compared to the 40 °C sample, which suggests that the 170 °C extract is chemically more complex than that at 40 °C. Nonetheless, the mean *m/z* values were comparable for all MS analyses (GALDI(−): 445.51 (40 °C)/426.80 (170 °C), GALDI(+): 406.42 (40 °C)/426.24 (170 °C)), which means that compounds of similar molecular weight were detected in both liquid extracts. The mass spectra of all GALDI-FT-ICR-MS analyses are presented in Supporting Information (SI) for this paper (see Figure S1).

**Figure 5:**
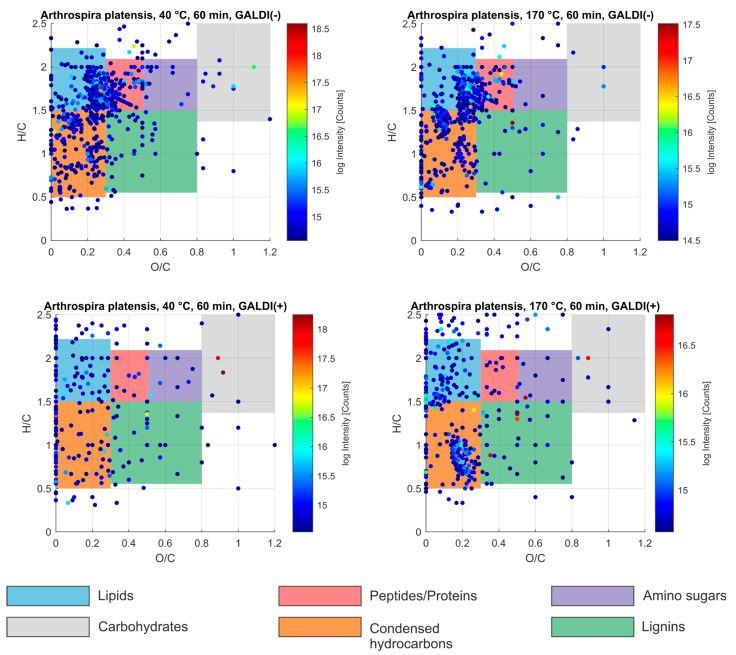
Van Krevelen plots for the microalgae (*A. platensis*) samples, which were analyzed by GALDI(−)- and GALDI(+)-FT-ICR-MS. The compound classes were assigned according to previous results [47,48] and are displayed color-coded, according to the description at the bottom. The observed intensity is presented logarithmically and color-coded (blue: low intensity, yellow: medium intensity, red: high intensity, see color bar).

To study the chemical complexity of both *A. platensis* liquid extracts, the mass spectrometric data sets of these samples were evaluated in the next step using van Krevelen plots. For these illustrations, the *O/C* and *H/C* ratios were calculated for each assigned molecular formula. Each molecular entity represents a single point in the plot. Specific regions within the *O/C* and *H/C* space correspond to distinct compound classes (e.g., lipids, sugars, peptides), enabling insights into the compositional diversity of complex samples. In the van Krevelen diagram, lignin-like structures are typically located within the range of *O/C* = 0.3–0.8 and *H/C* = 0.5–1.5. Condensed hydrocarbons share a similar *H/C* range but are generally restricted to lower *O/C* values (0–0.3). Other compound classes also occupy well-defined regions, such as lipids (*O/C* = 0–0.3, *H/C* = 1.6–2.3), peptides (*O/C* = 0.3–0.5, *H/C* = 1.5–2.1), amino sugars (*O/C* = 0.5–0.8, *H/C* = 1.5–2.1), and carbohydrates (*O/C* = 0.8–1.3, *H/C* = 1.4–2.4) [47,48]. The van Krevelen plots for both analyzed *A. platensis* liquid extracts are presented in Figure 5.

According to the van Krevelen plots, the GALDI(−)-MS data sets of both *A. platensis* samples are mainly dominated by condensed hydrocarbons, lipids, and amino sugars. In addition, lignin-like structures can also be assumed for both liquid extracts. Condensed hydrocarbons, lipids, and lignin-like molecules are also the most prevalent compounds in both samples according to the GALDI(+)-MS data sets. More in-depth structural evaluations based on the assigned molecular formulae to each ion in the GALDI-FT-ICR-MS data sets of both extracts can be found in Section S2 in the SI.

In this evaluation process, compounds that are typical for *A. platensis* microalgae could also be assumed in the analyzed liquid extracts. For instance, an ion (*m/z* 585.271859, C_33_H_38_N_4_O_6_, [M−H]^−^), possibly representing phycocyanobilin, a tetrapyrrole chromophoric molecule that is part of the phycocyanin of microalgae [49,50], was observable in the GALDI(−)-FT-ICR-MS data of the 40 °C *A. platensis* liquid extract (Intensity (*I*) = 1.52 ×·10^6^ Counts). Interestingly, this ion was not observed during the GALDI(−)-MS analysis of the 170 °C sample, which emphasizes that higher temperatures seem to significantly decrease the concentration of phycocyanin biomarkers in *A. platensis* [51].

Ions, that could indicate the presence of the phycocyanin discoloration agent biopterin-α-glucoside [27,28] were not detected in any FT-ICR-MS analysis. But the in-depth evaluation of the mass spectral data revealed that in both ion modes and for both liquid extracts, different ions were abundant (GALDI(−): [M−H]^−^, GALDI(+): [M+Na]^+^, [M+K]^+^), which suggests the presence of structurally similar molecules to biopterin-α-glucoside. Most of these different detected ions represent the same molecule with a molecular formula of C_14_H_19_N_5_O_7_. Compared to biopterin-α-glucoside (C_15_H_21_N_5_O_8_), the detected ionized molecule differs by CH_2_O in its molecular formula, which suggests that the molecule in question has a structure like biopterin-α-glucoside, but instead of a hexose, a pentose is bound to the biopterin backbone. Future tandem MS experiments can help to clarify the exact structure of this abundant molecular ion. The MS data of the 40 °C and 170 °C *A. platensis* liquid extracts showed an obvious intensity trend for the ions of this potential biopterin–pentoside. In both ion modes, the corresponding ions of C_14_H_19_N_5_O_7_ were more abundant in the 40 °C sample (GALDI(−): *I* = 1.19 × 10^8^ Counts ([M−H]^−^), GALDI(+): *I* = 2.27 × 10^7^ Counts ([M+Na]^+^), *I* = 2.16 × 10^7^ Counts ([M+K]^+^)) compared to the 170 °C extract (GALDI(−): *I* = 4.03 × 10^7^ Counts ([M−H]^−^), GALDI(+): *I* = 2.01 × 10^7^ Counts ([M+Na]^+^), *I* = 1.71 × 10^7^ Counts ([M+K]^+^)). Thus, the concentration of this potential phycocyanin discoloration agent also seems to decrease with increasing temperature. Furthermore, additional structurally similar compounds to biopterin-α-glucoside and biopterin–pentoside, which contain both nitrogen and oxygen (e.g., in heteroatomic classes N_4_O_1_–N_4_O_8_, N_5_O_4_–N_5_O_7_, N_6_O_5_–N_6_O_9_, N_7_O_4_–N_7_O_8_ and N_8_O_5_–N_8_O_8_), are assumable in both liquid extracts, if plots of the carbon number (*n_C_*) vs. the double bond equivalent (*DBE*) of the assigned molecular formulae are evaluated (see Figures S3 and S4) [52].

### 2.7. Particle Size Analysis

**Figure 6:**
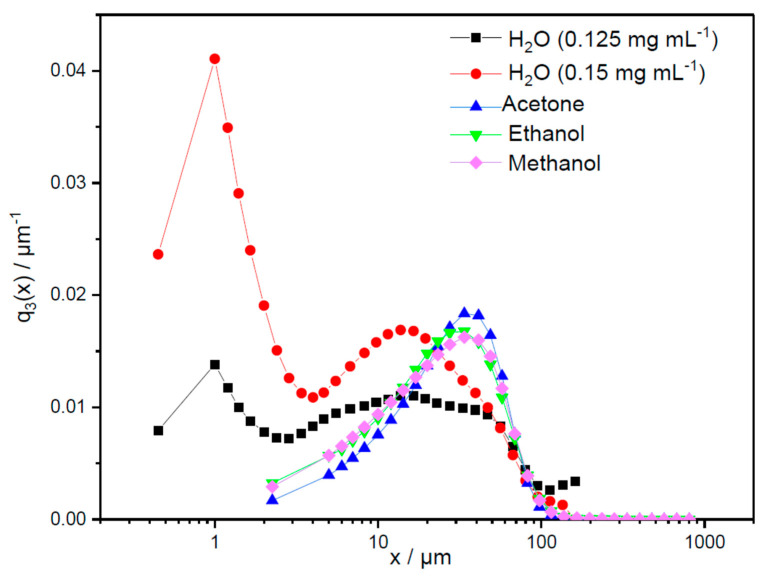
Volume-based density distribution (q_3_) in water, ethanol, methanol, and acetone shown over particle size diameter in µm.

**Figure 7:**
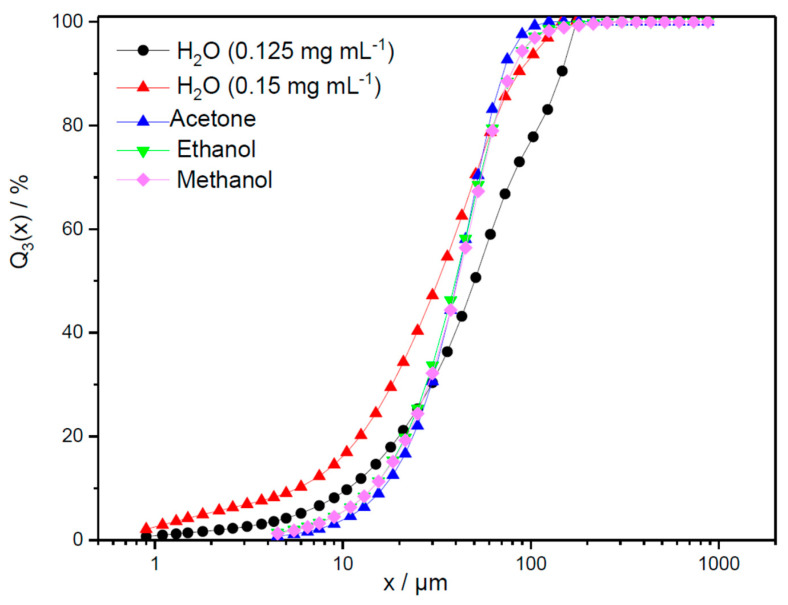
Cumulative volume distribution (Q_3_) in water, ethanol, methanol, and acetone shown over the particle diameter in µm.

Results for the particle size distribution are shown in Figure 6, presenting the density plots (q_3_), and Figure 7, presenting the cumulative sums (Q_3_). The modal particle sizes of the Millipore water samples with varying concentrations show similar values, namely $x_{mod1,water}=1\;µm$, $x_{mod2,0.125\frac{\mathrm{mg}}{\mathrm{mL}}water}=16.5\;µm,$ and $x_{mod2,0.15\frac{\mathrm{mg}}{\mathrm{mL}}water}=13.75\;µm$. 

Figure 6 illustrates the broader second peak of the low-concentration pigment sample (0.125 mg mL^−1^), with the cumulative sum shifted to the right. The broader peak observed in the 0.125 mg mL^−1^ sample indicates that the particle size distribution is more spread out with a wider range of particle sizes, whereas its shift to the right means the presence of large particles. This results in deviating median values for these two different concentrations in Millipore water, $x_{50,\;\;0.125\;\frac{mg}{mL}}=50.30\;µm$ and $x_{50,0.15\frac{mg}{mL}}=32.23\;µm$. Accordingly, a higher concentration in Millipore water in the given concentration range does not appear to enhance agglomeration. Since the measured size distributions of samples remained stable across all measurements, strong hydrophobic interactions and associated agglomeration during measurement time cannot be assumed. In contrast to the aqueous pigment solution, the pigment samples in acetone, ethanol, and methanol show similar monomodal distributions, with a modal value of 33.75 µm. These modal values are twice as large as the modal particle sizes of the aqueous pigment samples. Nevertheless, Figure 7 shows that the cumulative distributions of all three pigment samples in solvents fall between the distributions of the 0.125 mg mL^−1^ and 0.15 mg mL^−1^ aqueous samples, with median values of $x_{50,acetone}=40.58\;µm$, $x_{50,ethanol}=39.85\;µm$, and $x_{50,methanol}=41.00\;µm$.

This is because there is a significant number of particles below the modal value, broadly distributed over a wide particle size range, but fewer particles above the characteristic value. For the first 30% of the cumulative sum, the size distributions of the pigments in solvents show a similar behavior as the 0.125 mg mL^−1^ aqueous sample but with slightly rougher particle sizes. Later, the slope increases, with the last 20% of the cumulative sums being comparable to the 0.15 mg mL^−1^ sample that contained slightly finer particle sizes. The normalized widths of the distributions, $b=\frac{x_{90,i}-x_{10,i}}{x_{50,i}}$, show narrower distributions for the three pigment samples in solvents ($b_{acetone}=1.42$, $b_{ethanol}=1.62$, $b_{methanol}=1.57$) compared to the pigment samples in Millipore water ($b_{0.125\frac{\mathrm{mg}}{\mathrm{mL}}water}=1.42$, $b_{0.15\frac{\mathrm{mg}}{\mathrm{mL}}water}=1.62$). These results do not indicate a clear recommendation for the selection of the solvent to provide better stability with less pigment agglomeration. However, when acetone was used as a solvent for storing phycocyanin in this study, it resulted in the smallest distribution width, making it more advantageous than other solvents.

## 3. Discussion

This study aimed to investigate the effects of ultraviolet (UV) light irradiation on the pigment performance in the microwave-assisted extraction of *A. platensis*. The results demonstrate that the extraction conditions, including solvent type, temperature, and microwave power, significantly influenced the yield and stability of phycocyanin, the primary pigment of interest.

The choice of solvent played a crucial role in the extraction efficiency and stability of phycocyanin. Water, acetone, ethanol, and methanol were used as solvents, with water showing the highest yield of phycocyanin at 3.1 mg mL^−1^ at 30 °C. This aligns with previous studies that highlight the effectiveness of aqueous solutions in maintaining the integrity of phycocyanin during extraction [53,54].

In this study, the use of acetone with phycocyanin showed the narrowest particle size distribution that is desired by the textile and food industries. This, in combination with particle sizes less than 0.5 µm, can ensure uniformity and consistency in the release rate, which is crucial for maintaining the desired predictable and reproducible therapeutic or functional effect over time [55,56]. In addition, phycocyanin, encapsulated in acetone-based formulations, exhibits lower agglomeration compared to other solvents [57]. However, in this study, the differences in the particle size distributions among all solvents were minimal. 

In contrast to the nearly similar particle size distributions in all solvents, the stability of the pigment varied between solvents, with acetone and ethanol showing significant phase separation and discoloration following UV exposure. This suggests that while these solvents can extract phycocyanin, they may not be ideal for applications requiring high pigment stability. It also showed that the sensitivity of phycocyanin to UV light in ethanol and acetone can lead to chromophore degradation and loss of fluorescence. The addition of stabilizing agents or the use of UV-protective packaging for the phycocyanin transportation and storage could be potential strategies to mitigate this issue in practical applications [58,59].

Temperature and microwave power were critical parameters affecting the extraction process, with the impact of microwave power being particularly underexplored in the literature. This study showed that temperatures above 50 °C led to a decrease in phycocyanin yield and stability, corroborating earlier findings that high temperatures can denature phycocyanin. The optimal temperature for maintaining pigment integrity was identified as 45 °C, with a microwave power of 100 W. Increasing the power to 300 W at this temperature resulted in further pigment degradation, indicating that careful control of microwave power is necessary to prevent thermal damage to the pigment. This is also in line with the findings on the changes in pH and electrical conductivity, with significant changes in the conductivity of the extracts when the treatment temperature exceeded 50 °C. Although pH values remained relatively stable between 6 and 8 across the entire temperature range during microwave treatment of *A. platensis*, the conductivity of extracts decreased by nearly half in all solvents at temperatures above 50 °C, causing color changes.

FT-ICR-MS analysis revealed that the chemical complexity of the extracts increased with higher extraction temperatures. The presence of phycocyanobilin, a key chromophore in phycocyanin, can be assumed in extracts obtained at 40 °C but not at 170 °C, indicating thermal degradation at higher temperatures. The novelty of this study lies in the potential application of identified biopterin–pentosides, which are known for their antioxidant properties during UV exposure [60]. By leveraging the natural antioxidant properties of biopterin–pentosides, it may be possible to develop phycocyanin products that are more resistant to UV-induced degradation, thereby extending their shelf life and effectiveness. The characterization of biopterin–pentosides provides a new perspective and adds to the understanding of the molecular mechanisms involved in phycocyanin stabilization.

## 4. Materials and Methods

### 4.1. Chemicals and Materials

Dried *A. platensis* cells were obtained from AGROLAB LUFA GmbH (Kiel, Germany). The samples were comminuted using a Mixer Mill MM 200 (Retsch, Haan, Germany) with 0.5 mm zirconium beads for 10 min with an operational frequency of 20 Hz. The protein and carbohydrate contents of *A. platensis* were determined by AGROLAB following standards [61]. The ash and moisture contents were measured following the ASTM E871 and ASTM E1755. Ultimate analysis results (CHN) were acquired using a Thermo Flash Smart instrument (Thermo Fisher Scientific, Hindley Green, UK), as described in ASTM D5373-02. The oxygen content was calculated by the difference according to a previous study [62]. Ash compositional analysis was performed by inductively coupled plasma–optical emission spectrometry (ICP-OES) with prior microwave digestion, according to ASTM D6349-13 [63]. The Cl and I content in the ash was determined by first subjecting the sample to bomb combustion, followed by ion chromatography (IC), as suggested in ASTM D7359. Acetone, ethanol, and methanol were supplied by Sigma-Aldrich (Arklow, Ireland), with ≥99.8%. Pure water was obtained from the Synergy® (Millipore SAS, Darmstadt, Germany) UV purification system (water resistivity of 18.2 MΩ cm).

### 4.2. Microwave-Assisted Extraction 

Extraction experiments were conducted in 30 mL vials heated in a microwave reactor (CEM Corporation Discover 2.0, Matthews, North Carolina, USA). For each trial, 1.5 g of dried *A. platensis* (1.6 g, moist) was used and dispersed in water, acetone, ethanol, and methanol. The reaction mixture was rapidly heated (<2 min) to the maximum temperature (30, 40, 45, 50, 60, 70, 80, 90, or 100 °C) with 100 W under magnetic stirring of 300 rpm using conical stirrers. The reaction mixture was held at this temperature for 60 min, and then cooled down by compressed air to 50 °C. An additional study on the residence impact was performed at 45 °C within 30 or 60 min. The power was increased from 100 to 300 W at 45 °C and held for 60 min. Liquid extracts were filtered onto 0.2 µm PVDF membrane filters to remove cells and cell debris following previous research [16,64]. Then, the pigment solutions were dried using a rotary evaporator under vacuum at 40 °C and redispersed in 5 mL of selected solvent for further characterization on the same day the microwave extraction was performed. The pH values and electrical conductivity of the liquid extracts were determined accordingly. A pH electrode designed for use with non-aqueous solutions was used to measure pH values in acetone, ethanol, and methanol. The temperature was controlled and kept consistent during the measurements to avoid fluctuations in the presence of organic solvents.

### 4.3. Fluorescence Microscopy

The extract samples from microwave treatments in a wide temperature range were exposed to the UV light (20 mW cm^−2^; 365 nm) for 3 min to investigate the light sensitivity in various solvents. In addition, extracts from microwave treatment at 50 °C were exposed to UV light in tetrahydrofuran (THF, Sigma-Aldrich, Arklow, Ireland) and dimethyl carbonate (DMC, Sigma-Aldrich, Arklow, Ireland), aiming to show the impact of polar and non-polar solvents. 

The dispersed pigments in acetone, ethanol, methanol, tetrahydrofuran, and dimethyl carbonate were observed using a Zeiss Axio Lab.A1 (Zeiss, Oberkochen, Germany) in fluorescent microscopy mode. Dispersed pigment samples in solvents were placed on the microscopic glass and magnified until the structures became visible. Photos and macroscopic close-up pictures were taken using the Axiocam 305 color camera (Zeiss, Oberkochen, Germany) with 5 megapixels and a 2/3-inch sensor at the excitation wavelength of 365 nm. The pictures were processed using the free software ZEN 2 core v2.4.

### 4.4. UV-Vis Spectroscopy

To conduct UV-Vis spectroscopy, dispersed pigments in various solvents were diluted using ratios (2:1; 1:1; 1:2; 1:29) in corresponding solvents. For the calibration (2, 5, 7.5, 10, 12.5, or 15 mg mL^−1^), standards were supplied by Algen Markt (Gießen, Germany). The spectra were measured by a SPECORD Plus spectrometer (Analytik Jena, Jena, Germany) in the wavelength range from 185 to 1200 nm, operated at a resolution of 5 nm using a quartz cuvette with a path length of 1 cm (quartz suprasil, Hellma Analytics, Müllheim, Germany). 

The concentration of phycocyanin (PC) in mg mL^−1^ was calculated from the optical densities (A) at 617 nm and 652 nm using an equation for extracts in water, acetone, ethanol, and methanol [65]:

$$PC\;\left(\frac{\mathrm{mg}}{\mathrm{mL}}\right)=\frac{A_{617}-(0.474\;A_{652})}{5.34}$$

The uncertainty was calculated based on absorption measurements taken in at least triplicate. The calibration curves are shown in the Supplementary Materials (Figures S7–S10).

### 4.5. FT-ICR-MS Analyses and Data Processing

The liquid extracts of two *A. platensis* samples, prepared at 40 °C and 170 °C for 60 min using a power of 100 W, were analyzed using a Bruker Daltonics solariX 15 T FT-ICR-MS (Bremen, Germany). Ion generation from these extracts was achieved through graphite-assisted laser desorption/ionization (GALDI), conducted in negative (GALDI(−)) and positive (GALDI(+)) ion mode [66]. To prepare the samples for GALDI-FT-ICR-MS analysis, 30 mg of high-purity graphite (Micro to Nano, purity ≥ 99.9%, particle size 5 μm) was weighed into an amber glass vial. This was followed by the addition of 100 µL of the liquid extract and 100 µL of methanol (Merck Supelco, LiChrosolv, Arklow, Ireland, purity ≥ 99.8%). The resulting suspension was sonicated for 10 min, and 1.2 μL of each suspension was spotted onto a MALDI steel target and left to air dry.

The FT-ICR-MS was equipped with a MALDI source featuring a Smart Beam II laser (frequency-tripled Nd:YAG laser, *λ* = 355 nm, pulse duration of 3 ns, pulse energy of 500 μJ, peak power of 170 kW, and average power of 1.5 W). GALDI analyses employed an ultra-large laser focus (spot size of ~250 μm) with 20 laser shots per spot. The plate offset and deflector plate voltages were set to −60 V and −180 V for GALDI(-) and +40 V and +200 V for GALDI(+), with a laser frequency of 500 Hz. Laser powers ranged from 17–20%, depending on the sample and ion mode.

For the FT-ICR-MS analyses, a scan range of *m/z* 153.49–2000.00 was used, with a Q1 mass of *m/z* 170. Data sets were collected at a size of 8 M, with both liquid extracts analyzed over 64 scans. All analyses achieved a resolving power of *R* = 800,000 at *m/z* 400.

Peak picking, calibration, and molecular formula assignment were performed using Bruker Daltonics DataAnalysis 5.0 (SR 1). The mass calibration for the GALDI-FT-ICR-MS experiments involved a two-step process. Initially, pre-existing calibration lists were applied for the first round of internal calibration. Molecular formulae were then derived from the mass spectra, and these formulae were utilized to generate a refined internal calibration list containing molecular ions representative of the two *A. platensis* samples under analysis. Peaks with a signal-to-noise ratio (*s/n*) ≥ 10 were assigned molecular formulae. The mass deviation from theoretical values was restricted to ≤ 0.3 ppm, and the assigned formulae adhered to a specific elemental composition (C_c_H_h_N_n_O_o_S_s_Na_na_K_k_: *c* = unlimited, *h* = unlimited, 0 ≤ *n* ≤ 10, *o* = unlimited, 0 ≤ *s* ≤ 2, 0 ≤ *na* ≤ 3, 0 ≤ *k* ≤ 1). The resulting peak and molecular formula lists were exported to MATLAB R2024b (MathWorks), where they underwent additional processing and visualization through custom scripts for blank correction and molecular formula filtering. The filtering of molecular formulae followed established criteria, including double bond equivalent (*DBE*) ≥ 0, 0.3 ≤ *H/C* ≤ 2.5, *O/C* ≤ 1.2, *N/C* ≤ 1.0, and *S/C* ≤ 1.0 [67,68].

### 4.6. Particle Size Analysis of Pigments

Particle size distributions were measured using the laser diffractor Helos (Sympatec, Clausthal-Zellerfeld, Germany) with CUVETTE and measurement range mode R3 (0.5–175 µm), respectively R5 (4.5–875 µm). Since slight sedimentation of the samples was visible, the magnetic stirrer inside CUVETTE was set to about 10% to avoid inconsistent measurements caused by instabilities. For each sample, a minimum of six measurements were carried out, each including a background and sample measurement, respectively. An example demonstrating the reproducibility of the measurements is shown in the Supplementary Materials (Figure S6). Two samples with different pigment concentrations were investigated in deionized (DI) Millipore water, namely 0.125 mg mL^−1^ and 0.15 mg mL^−1^. Measurements in acetone (purity > 99.5%, Carl Roth, Karlsruhe, Germany), isopropanol (purity > 99.5%, Carl Roth, Karlsruhe, Germany), and ethanol (purity > 99.5%, Carl Roth, Karlsruhe, Germany) were taken using pigment concentration samples of 0.15 mg mL^−1^. The respective liquid was placed in the CUVETTE, and the samples (40 mL) were transferred so that the same dilution conditions prevailed for all samples.

## 5. Conclusions

This study investigated the effects of UV light irradiation on pigment performance in the microwave-assisted extraction of *A. platensis*, focusing on phycocyanin. The results show that water is the most effective solvent, yielding the highest phycocyanin concentration and better particle stability compared to organic solvents. Acetone and ethanol, despite their extraction capabilities and uniform phycocyanin particle sizes, showed significant phase separation and discoloration upon UV exposure. Optimal extraction conditions were identified as 45 °C and 100 W microwave power, with higher temperatures leading to pigment degradation. FT-ICR-MS analysis revealed the presence of phycocyanobilin and proteins as a biopterin–pentoside complex at 40 °C but not at 170 °C, indicating thermal degradation. This discovery provides new insights into the molecular mechanisms of phycocyanin stabilization. Future research should focus on refining extraction parameters and exploring additional stabilizing agents to further enhance phycocyanin stability and functionality.

## Figures and Tables

**Table 1:** Proximate, ultimate, and ash compositional analysis of dried *A. platensis*.

Moisture, wt.% (as received)	7.1
Ash, wt.% (as received)	9.3
Protein g/100 g	64.7
Carbohydrate g/100 g	8.6
Ultimate analysis % (on a dry basis)	
Carbon	45.9
Hydrogen	6.2
Oxygen	25.4
Nitrogen	6.1
Ash composition in mg kg^−1^	
Cl	17,200
I	900
S	27,000
Al	20,600
Ca	11,600
Cr	30
Cu	70
Fe	10,000
K	6000
Mg	4000
Mn	200
Na	7600
Ni	5
Zn	300
As	8
Hg	10
Mo	11
P	14,000
P	14,000
Se	0

**Table 2:** Concentrations of phycocyanin in mg mL^−1^ in extracts from microwave treatment ranging in temperature from 30 to 70 °C.

Solvent	Phycocyanin Concentrations (mg mL^−1^) with Standard Deviation							
	30 °C	40 °C	45 °C	45 °C	45 °C	50 °C	60 °C	70 °C
	60 min	60 min	60 min	30 min	60 min	60 min	60 min	60 min
	100 W	100 W	100 W	100 W	300 W	100 W	100 W	100 W
Water	3.1 ± 0.007	1.9 ± 0.016	2.1 ± 0.01	1.8 ± 0.005	1.7 ± 0.007	0.7 ± 0.017	0.4 ± 0.005	0.3 ± 0.010
Acetone	2.2 ± 0.017	2.1 ± 0.016	1.7 ± 0.009	1.8 ± 0.012	1.7 ± 0.004	2.1 ± 0.126	0.6 ± 0.009	0.4 ± 0.009
Ethanol	2.1 ± 0.015	2.0 ± 0.009	1.8 ± 0.008	1.9 ± 0.008	1.8 ± 0.012	1.7 ± 0.007	0.6 ± 0.019	0.5 ± 0.004
Methanol	2.0 ± 0.006	1.8 ± 0.006	1.9± 0.011	1.9 ± 0.016	1.7 ± 0.005	1.9 ± 0.009	0.5 ± 0.008	0.4 ± 0.009

## Data Availability

The data presented in this study are available on request from the corresponding author.

## Supplementary Materials

The following supporting information can be downloaded at https://www.mdpi.com/article/10.3390/md23100391/s1: Figure S1: GALDI(−)- and GALDI(+)-FT-ICR-MS mass spectra for the *A. platensis* liquid extracts, which were prepared at 40 °C and 170 °C for 60 min. Figure S2: Comparison of assigned number of molecular formulae (A) and relative abundancies (B) to the FT-ICR-MS data sets of the microalgae liquid extracts, prepared at 40 °C and 170 °C for 60 min, for compound classes N1–N6, N1O1–N1O4, N2O1–N2O5, N3O1–N3O5, N4O1–N4O8, N5O4–N5O7, N6O5–N6O9, N7O4–N7O8, N8O5–N8O8, S1–S2, S1O1–S1O4, and O1–O14. Figure S3: nC-DBE plots for compound classes N4O1–N4O4 of the GALDI-FT-ICR-MS data of the two analyzed *A. platensis* samples. The observed intensity is presented logarithmically and color-coded (blue: low intensity, yellow: medium intensity, red: high intensity). Figure S4: nC-DBE plots for compound classes N5O4–N5O7 of the GALDI-FT-ICR-MS data of the two analyzed *A. platensis* samples. The observed intensity is presented logarithmically and color-coded (blue: low intensity, yellow: medium intensity, red: high intensity). Figure S5: nC-DBE plots for compound classes O9–O12 of the GALDI-FT-ICR-MS data of the two analyzed *A. platensis* samples. The observed intensity is presented logarithmically and color-coded (blue: low intensity, yellow: medium intensity, red: high intensity). Figure S6: Cumulative volume distribution of phycocyanin in methanol shown over the particle diameter in µm measured seven times. Figure S7: UV-Vis calibration curve of the phycocyanin standard in Millipore water through the linear fitting of measured points. Figure S8: UV-Vis calibration curve of the phycocyanin standard in ethanol through the linear fitting of measured points. Figure S9: UV-Vis calibration curve of the phycocyanin standard in acetone through the linear fitting of measured points. Figure S10: UV-Vis calibration curve of the phycocyanin standard in methanol through the linear fitting of measured points.
