# Supplementary material for: The Effect of Ultraviolet Light Irradiation on Pigment Performance in Microwave-Assisted Extraction of Arthrospira platensis
Source: Mar Drugs. 2025 Sep 30;23(10):391. doi: 10.3390/md23100391 (PMC12565327; doi:10.3390/md23100391)
Supplement: Supplementary file 1 [file marinedrugs-23-00391-s001.zip › marinedrugs-3875894-supplementary.pdf]

The Effect of Ultraviolet Light Irradiation on Pigment Performance in Microwave-assisted Extraction of *Arthrospira platensis*

- SUPPORTING INFORMATION -

S1. Mass spectra of the two Arthrospira platensis samples analyzed by GALDI-FT-ICR-MS

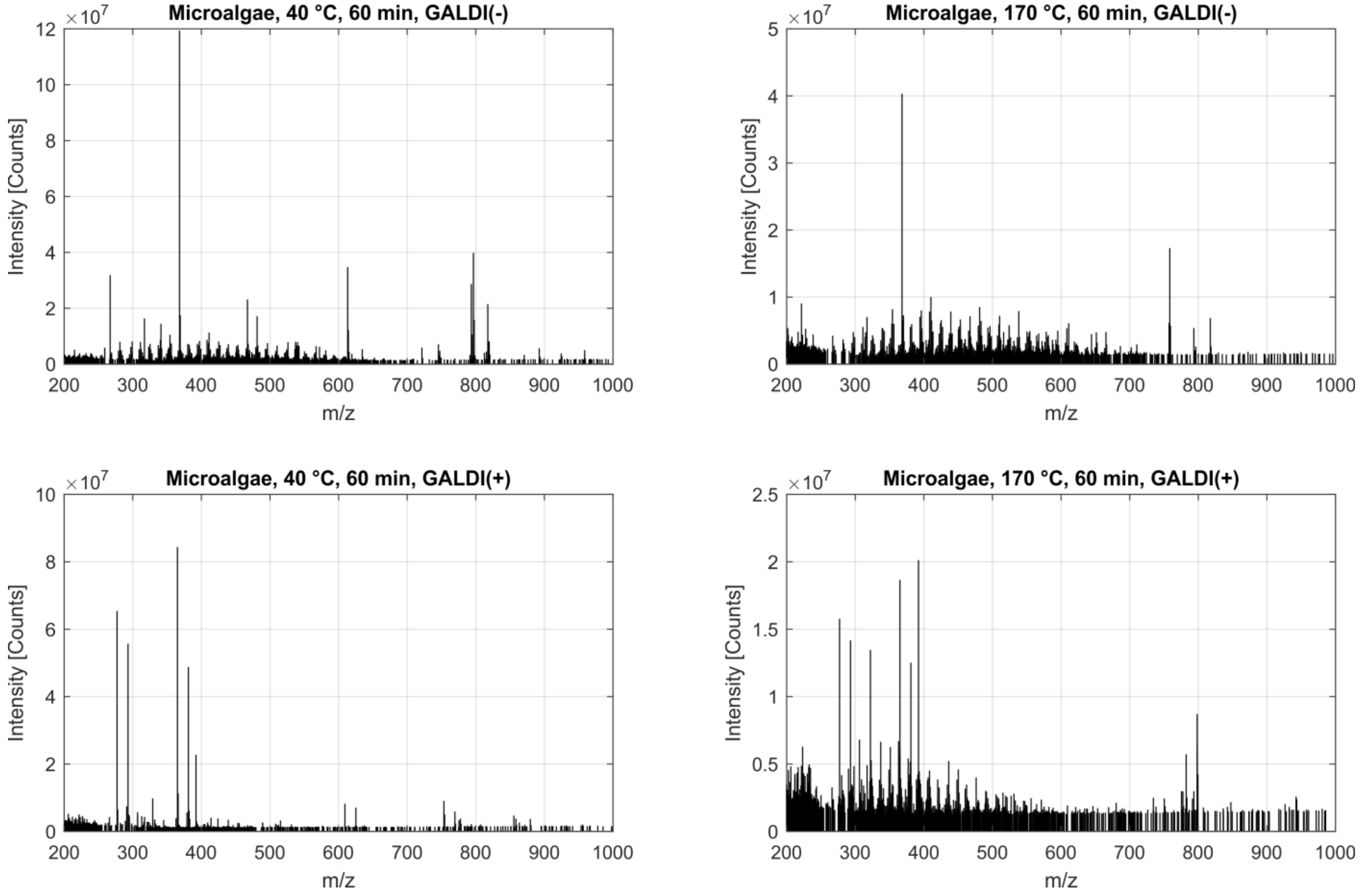

Figure S1: GALDI(-)- and GALDI(+)-FT-ICR-MS mass spectra for the Arthrospira platensis liquid extracts, which were prepared at 40°C and 170°C for 60 min.

S2. Molecular formula evaluation of the GALDI-FT-ICR-MS data sets

Detailed insights into the various compounds and compound classes detected by GALDI-FT-ICR-MS can be obtained by clustering the assigned molecular formulae of these molecules into heteroatomic classes, based on the number of oxygen (O), nitrogen (N) and/or sulfur (S) atoms. These heteroatomic classes are typically analyzed in terms of the total number of assigned molecular formulae and the relative abundance of each heteroatomic class. Figure S2 presents visualizations of these metrics for the compound classes N_1 – N_6, N_1O_1 – N_1O_4, N_2O_1 – N_2O_5, N_3O_1 – N_3O_5, N_4O_1 – N_4O_8, N_5O_4 – N_5O_7, N_6O_5 – N_6O_9, N_7O_4 – N_7O_8, N_8O_5 – N_8O_8, S_1 – S_2, S_1O_1 – S_1O_4 and O_1 – O_{14}, which exhibited the highest numbers of assigned molecular formulae across all analyzed samples. Molecules, which contained only nitrogen and no other heteroatoms (N_1 – N_6), were mainly assigned to the GALDI(+)-MS data sets of the two microalgae samples. This suggests that these N-functionalized molecules are mostly compounds with basic functionalities, as these are easier to ionize (e.g., by protonation) in positive ion mode. In comparison, nitrogen- and oxygen-containing compounds (N_1O_1 – N_1O_4, N_2O_1 – N_2O_5, N_3O_1 – N_3O_5, N_4O_1 – N_4O_8, N_5O_4 – N_5O_7, N_6O_5 – N_6O_9, N_7O_4 – N_7O_8, N_8O_5 – N_8O_8) are found in both ion modes for the *Arthrospira platensis* samples, even though higher numbers and abundancies for compound classes N_4O_1 – N_4O_8, N_5O_4 – N_5O_7, N_6O_5 – N_6O_9, N_7O_4 – N_7O_8 and N_8O_5 – N_8O_8 are observable for the GALDI(-)-MS data sets. Thus, molecules in these compound classes seem to possess acidic substructures that are more easily deprotonated in the ionization process. Sulfur- (S_1 – S_2) as well as sulfur-oxygen-containing compounds (S_1O_1 – S_1O_4) are equally observable in both ion modes. Only small amounts of purely oxygen-functionalized molecules are assumable in the GALDI(-)- and GALDI(+)-FT-ICR-MS data sets, especially in the heteroatomic classes O_1 – O_{10}. Compounds that contain 11 up to 14 oxygen atoms (O_{11} – O_{14}) are mainly observable in the negative ion mode.

Structural information about the various heteroatomic classes and the compounds within them can be derived through visualizations of the carbon number (n_C) versus the double bond equivalent (*DBE*). Both properties are determined from the molecular formula assigned to each ion. The n_C value corresponds to the number of carbon atoms, while the *DBE* is calculated based on the assigned number of carbon, hydrogen and nitrogen atoms. The *DBE* reflects the total number of double bonds and rings in the molecular structure, whereas the n_C provides insights into the degree of alkylation within a compound class.

In Figure S3 – S5, exemplary n_C-*DBE* plots for heteroatomic classes N_4O_1 – N_4O_4, N_5O_4 – N_5O_7 and O_9 – O_{12} are illustrated. Especially according to the plots of the nitrogen- and oxygen-containing classes (N_4O_1 – N_4O_4 and N_5O_4 – N_5O_7, see Figures S3 – S4), unsaturated/aromatic molecules with n_C = 10 – 25 and *DBE* = 3 – 11 are assumable. These compounds are nearly exclusively observable in the visualizations of the GALDI(-)-MS data sets of both *Arthrospira platensis* liquid extracts, which suggest that at least one

deprotonable functionality (e.g., hydroxy or carboxy group) might be present in the structure of these ionized molecules. In conjunction with the high number of nitrogen and oxygen atoms assigned to these molecules, compounds with a similar structural composition as the phycocyanin discoloration agent biopterin-α-glucoside ($C_{15}H_{21}N_5O_8$) and the proposed biopterin-pentoside ($C_{14}H_{19}N_5O_7$) can be assumed. The n_C-DBE plots of heteroatomic classes O_9 – O_{12} (see Figure S5) are mainly dominated by unsaturated molecules (DBE = 1 – 3) with n_C values between 19 and 30. Hence, multiple oxygen-functionalized (hydroxy, ether, aldehyde, carbonyl, ester, carboxyl) aliphatic structures are also most likely present in the two microalgae samples.

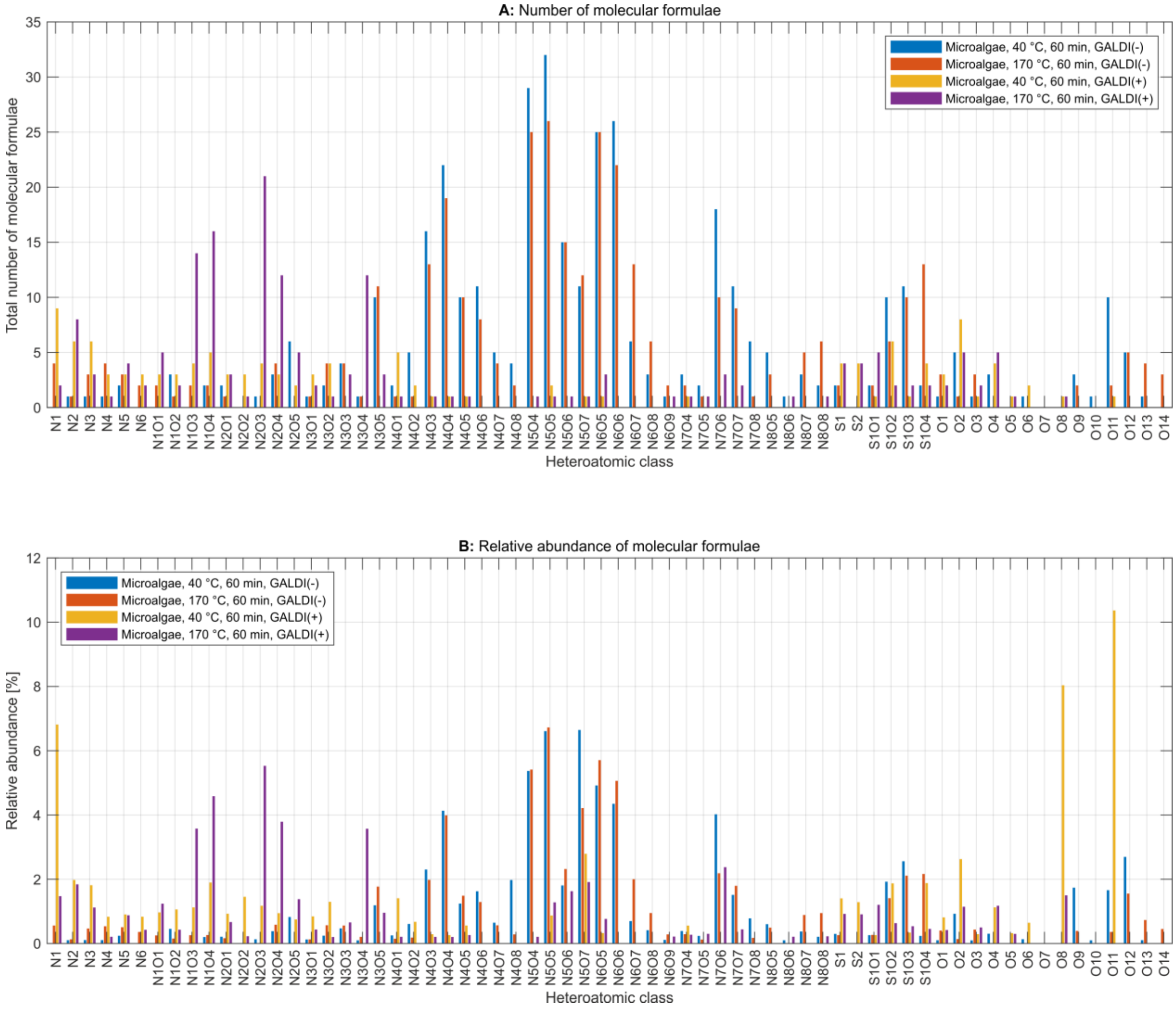

Figure S2: Comparison of assigned number of molecular formulae (S2A) and relative abundancies (S2B) to the FT-ICR-MS data sets of the microalgae liquid extracts, prepared at 40°C and 170°C for 60 min, for compound classes N_1 – N_6, N_1O_1 – N_1O_4, N_2O_1 – N_2O_5, N_3O_1 – N_3O_5, N_4O_1 – N_4O_8, N_5O_4 – N_5O_7, N_6O_5 – N_6O_9, N_7O_4 – N_7O_8, N_8O_5 – N_8O_8, S_1 – S_2, S_1O_1 – S_1O_4 and O_1 – O_{14}.

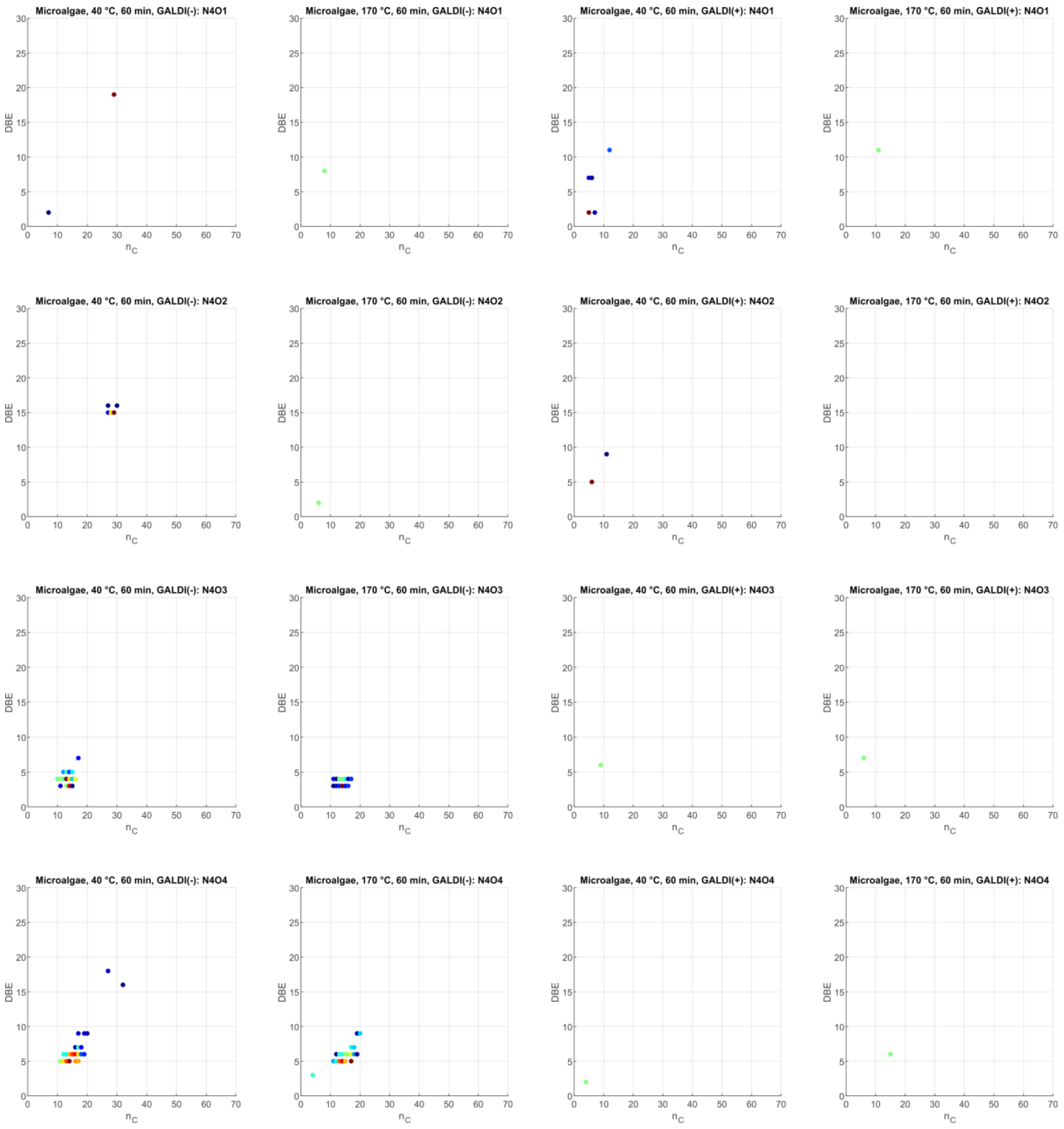

Figure S3: n_C-DBE plots for compound classes N_4O_1 – N_4O_4 of the GALDI-FT-ICR-MS data of the two analyzed Arthrospira platensis samples. The observed intensity is presented logarithmically and color-coded (blue: low intensity, yellow: medium intensity, red: high intensity).

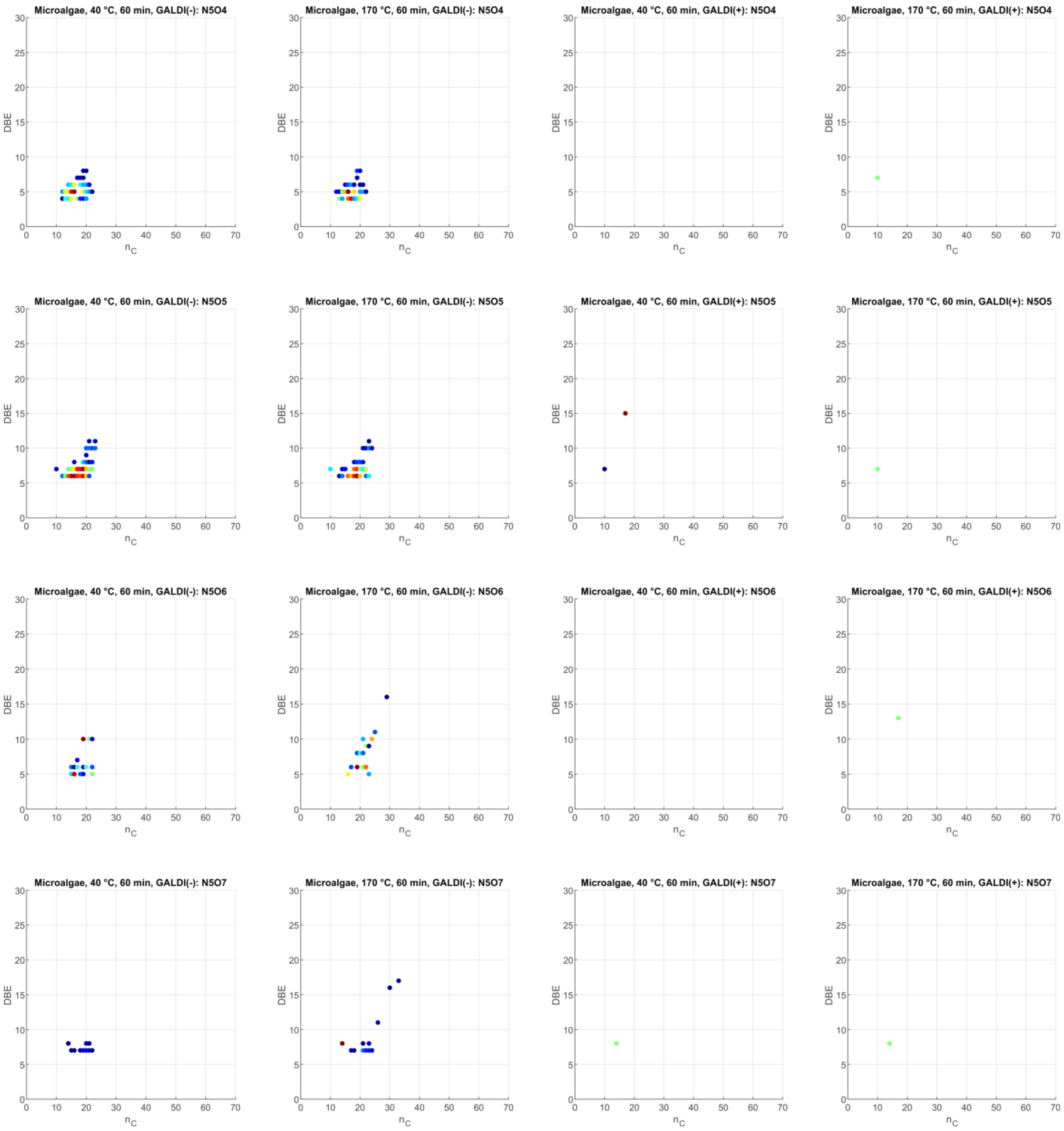

Figure S4: n_C-DBE plots for compound classes N_5O_4 – N_5O_7 of the GALDI-FT-ICR-MS data of the two analyzed Arthrospira platensis samples. The observed intensity is presented logarithmically and color-coded (blue: low intensity, yellow: medium intensity, red: high intensity).

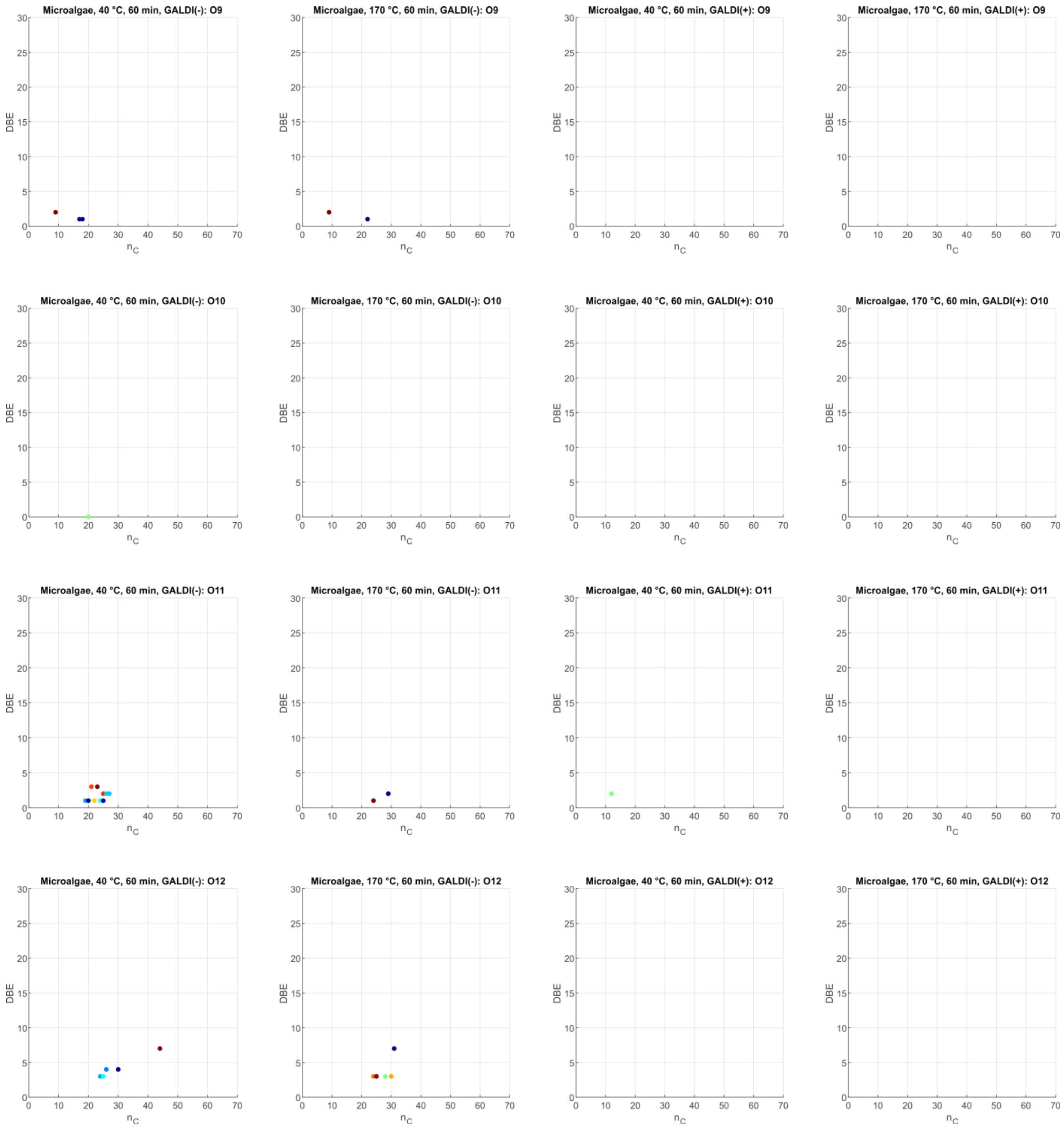

Figure S5: n_C-DBE plots for compound classes O_9 – O_{12} of the GALDI-FT-ICR-MS data of the two analyzed Arthrospira platensis samples. The observed intensity is presented logarithmically and color-coded (blue: low intensity, yellow: medium intensity, red: high intensity).

S3. Particle size analysis

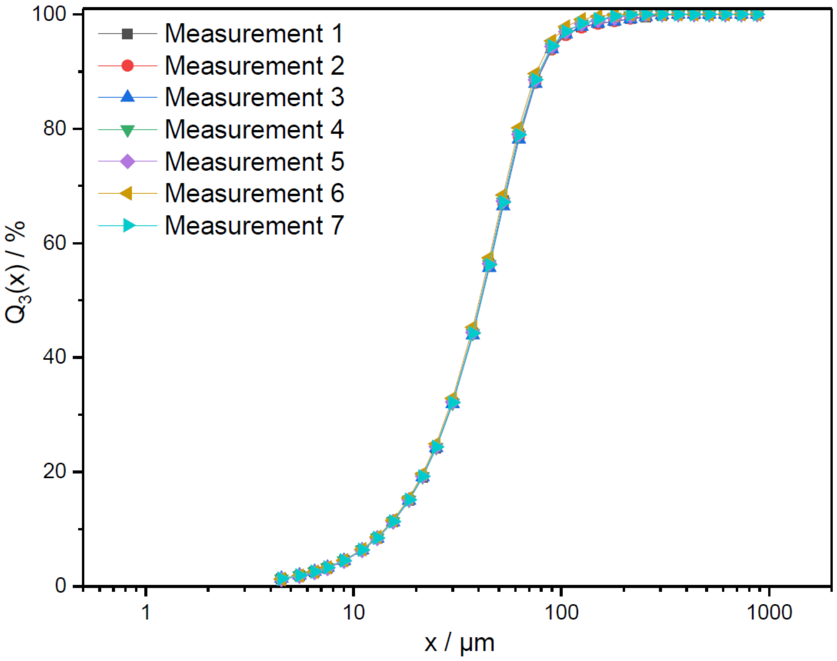

Figure S6: Cumulative volume distribution of phycocyanin in methanol shown over the particle diameter in µm measured seven times.

S4. Calibration curves of UV-Vis phycocyanin's analysis

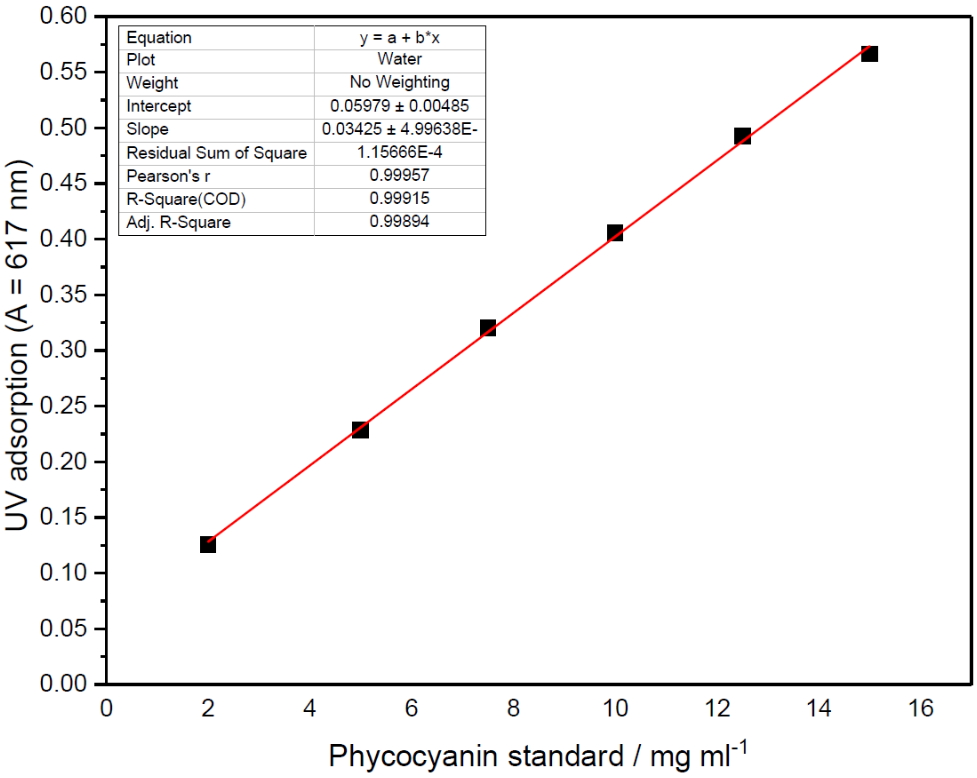

Figure S7: UV-Vis calibration curve of the phycocyanin standard in Millipore water through the linear fitting of measured points.

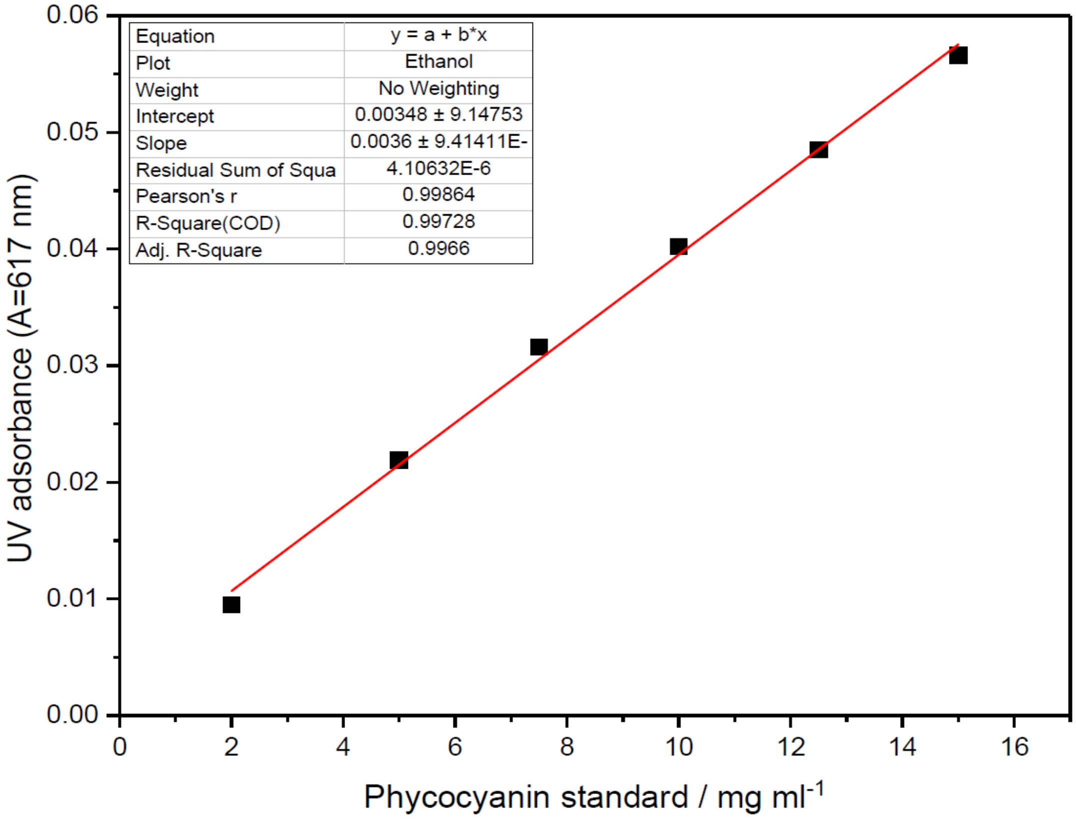

Figure S8: UV-Vis calibration curve of the phycocyanin standard in ethanol through the linear fitting of measured points.

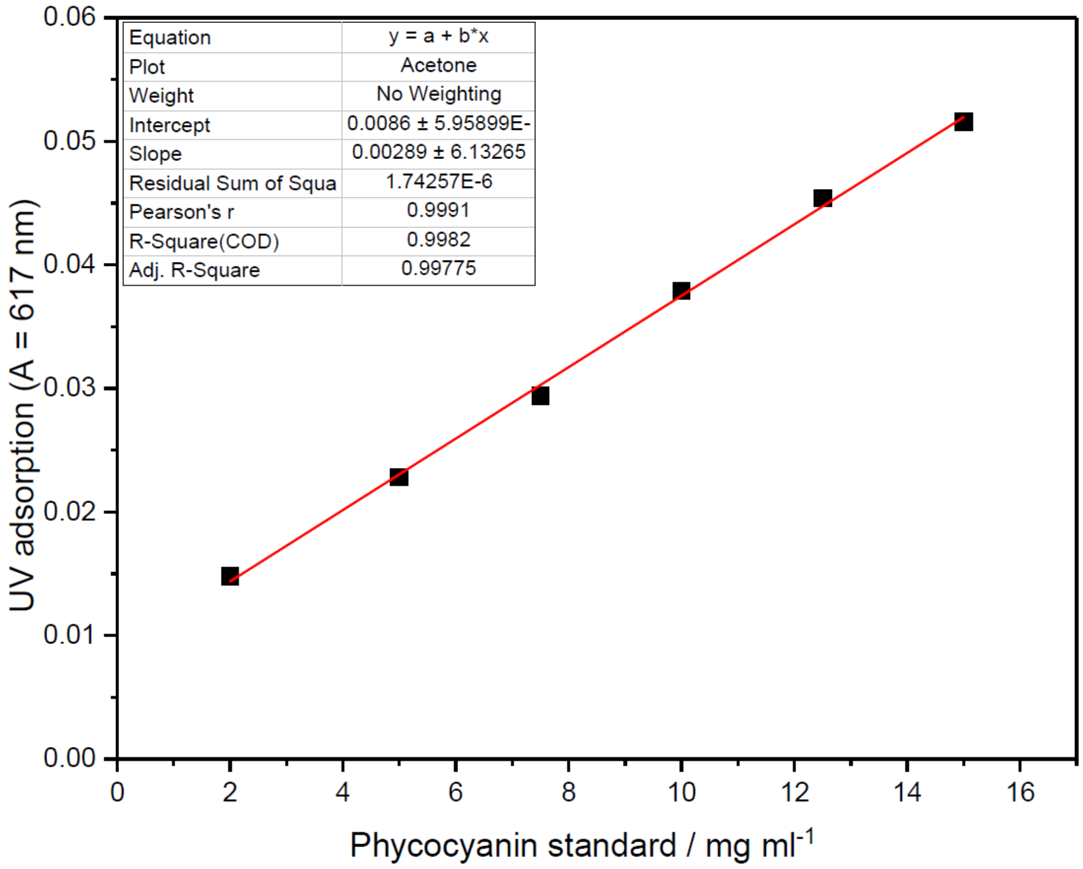

Figure S9: UV-Vis Calibration curve of the phycocyanin standard in acetone through the linear fitting of measured points.

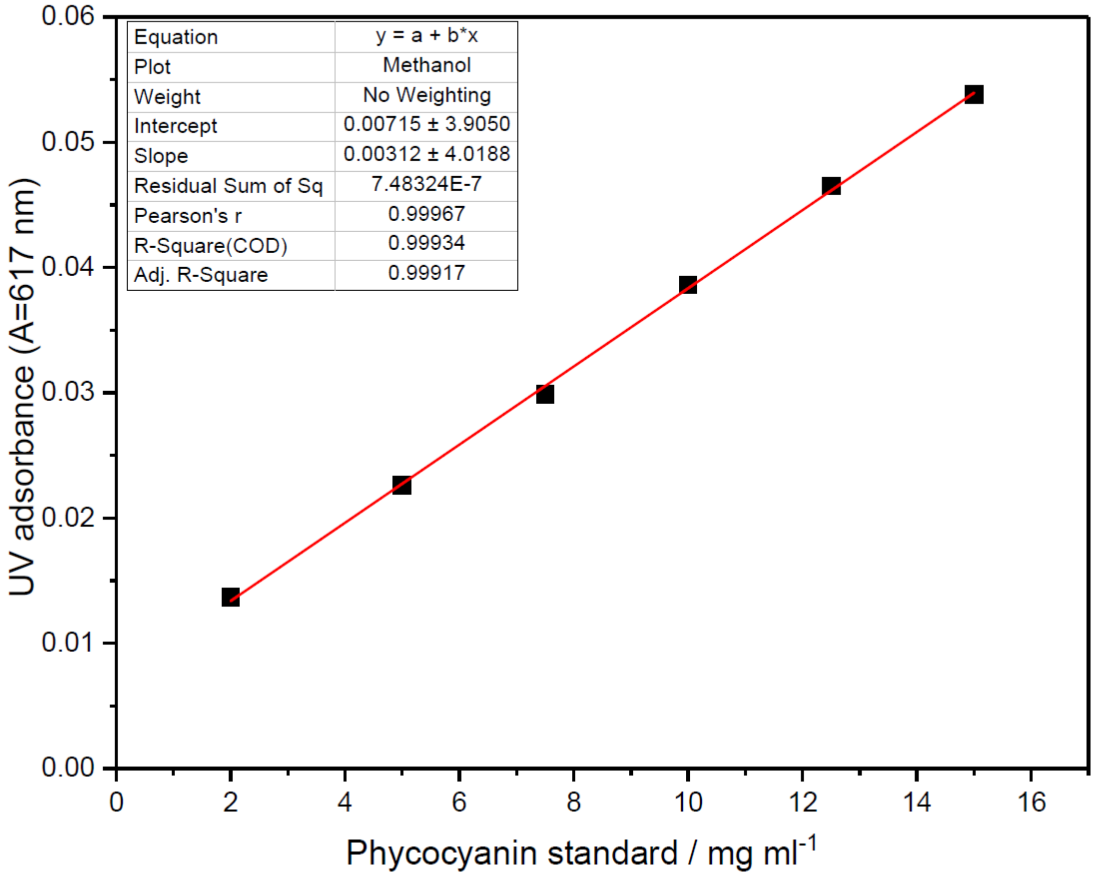

Figure S10: UV-Vis Calibration curve of the phycocyanin standard in methanol through the linear fitting of measured points.
